# Plant-Based Dietary Patterns versus Meat Consumption and Prevalence of Impaired Glucose Intolerance and Diabetes Mellitus: A Cross-Sectional Study in Australian Women

**DOI:** 10.3390/nu14194152

**Published:** 2022-10-06

**Authors:** Courtney L. Baleato, Jessica J. A. Ferguson, Christopher Oldmeadow, Gita D. Mishra, Manohar L. Garg

**Affiliations:** 1 Nutraceuticals Research Program, School of Biomedical Sciences & Pharmacy, University of Newcastle, Callaghan, NSW 2308, Australia; 2 Food & Nutrition Program, Hunter Medical Research Institute, New Lambton Heights, NSW 2305, Australia; 3 Clinical Research Design, Information Technology and Statistical Support Unit, Hunter Medical Research Institute, New Lambton Heights, NSW 2305, Australia; 4 School of Public Health, The University of Queensland, Herston, QLD 4006, Australia

**Keywords:** plant-based diets, diabetes, impaired glucose tolerance, women, dietary patterns

## Abstract

This study aimed to compare the prevalence of impaired glucose tolerance (IGT) and diabetes mellitus (DM) among Australian women following plant-based diets (PBD) compared to regular meat eaters. A cross sectional analysis of the mid-aged cohort (1946–1951) of the Australian Longitudinal Study on Women’s Health was conducted on completers of Survey 7 in 2013 with complete FFQ data available (*n* = 9102). Dietary patterns were categorized as PBD (vegan, lacto-ovo vegetarian, pesco-vegetarian, semi-vegetarian) and regular meat eaters. Meat eaters were further categorized into high and low consumption and outcomes included self-reported prevalence of IGT and DM. Participants were identified as regular meat eaters (*n* = 8937) and PBD (*n* = 175). Prevalence of IGT was lower in PBD (0–1.2%) compared to regular meat eaters (9.1%). Consolidation of PBD to a single group (vegetarians) indicated a lower prevalence of DM in vegetarians compared to regular meat eaters (3.9% vs. 9.1%). Women consuming meat daily/multiple times per day had significantly higher odds of IGT (OR 1.5, 95%CI 1.1 to 2.1, *p* = 0.02). Individuals consuming processed meat daily/multiple times per day had significantly higher odds of DM compared to those consuming less than daily (Odds ratio (OR) 1.7, 95% confidence interval (CI) 1.3 to 2.3, *p* < 0.0001). After adjustment for covariates, statistical significance was lost largely due to the addition of BMI to the model. Prevalence of IGT and DM were lower in women following PBD and higher in high consumers of meat and processed meat. The relationship between meat consumption and IGT/diabetes status appears to be mediated, at least in part, by an increase in body mass index (BMI). Future studies are warranted to investigate the mechanisms and other lifestyle factors underpinning the association between high meat consumption and increased risk of IGT and DM.

## 1. Introduction

Diabetes Mellitus (DM) is a serious global public health concern with significant negative impacts on quality of life (QOL), morbidity, and mortality [1,2]. In 2019, diabetes was the ninth leading cause of death with 1.5 million deaths estimated to be directly caused by diabetes worldwide [3]. In Australia, type 2 diabetes (T2D) impacts nearly 5% of the total population, affecting over one million adults in 2016–2017 and contributing significantly to Australia’s burden of disease with a cost to the health care system of AUD 2.7 billion [4]. Risk factors for T2D vary and can be categorized as non-modifiable including age, race, ethnicity, genetics, [5,6] and modifiable including physical inactivity, elevated BMI, high body fat composition, smoking, alcohol consumption, and poor dietary choices [1,5,6]. Onset and experience of T2D in midlife (˃45 years) have much higher prevalence rates than younger age groups with diagnosis more than doubling in the last 2 decades in the over 65-year age group [4,7]. Impaired glucose tolerance (IGT) or “pre-diabetes” is diagnosed by elevated blood glucose levels that are not yet as high as the diagnostic criteria for T2D. IGT is recognized as a significant risk factor for developing T2DM and currently impacts approximately two million Australian adults [8].

Diets that are high in saturated fats and refined carbohydrates and low in fiber have been linked to increased visceral fat and higher waist circumference which are well-established risk factors for IGT and T2D [5,6,9,10,11]. Whilst general management recommendations for T2D include increased physical activity, cessation of smoking, and oral glucose-lowering medications, Diabetes Australia recognizes dietary interventions for sustained weight loss as the most effective approach to delay or halt the onset or progression of IGT and T2D [8,12]. Plant-based diets (PBD) are gaining traction across the globe out of concern for health, animal welfare and environmental sustainability [13,14]. Current Western dietary patterns are often energy dense, nutrient poor and low in fruit and vegetable consumption which are contributing to the rising rates of chronic illnesses like T2D, cardiovascular disease and cancer. PBDs have been studied for their potential preventative and therapeutic effects on T2D and have even been shown to be more beneficial than medication for disease management [15,16].

PBD are characterized by the emphasis on fruits, vegetables, grains, seeds, nuts, and legumes and generally exclude or limit animal flesh and animal products such as red meat, poultry, fish, dairy, and eggs [17,18]. It has been reported that diets rich in plant-based foods may provide more protection for the prevention of diet-related chronic diseases including T2D than diets that include animal-based foods [9]. Findings from randomized controlled trials (RCTs) indicate that healthful PBD may enhance glycemic control, improve blood lipid profiles, reduce central adiposity and reduce circulating inflammatory mediators [16,19]. A cohort study (*n* = 200, 727), along with a study involving T2D (*n* = 13) and a systematic review and meta-analysis of PBDs and weight status in individuals with T2D (*n* = 353) indicate that the combined high fiber content and low saturated fat of many PBD may assist in reducing/maintaining bodyweight and therefore may further reduce the risk of T2D [19,20,21]. Long-term adherence to a vegetarian diet has been associated with a 20–74% reduced risk of developing T2D [16,20]. This reduced risk has been identified in multiple studies across diverse population groups including postmenopausal overweight women, Seventh Day Adventists [22,23], American nurses and health care professionals [20], and non-diabetic normal-weight men [16].

Conversely, diets high in red and processed meats, as well as animal-derived saturated fats, have been linked with an increased risk of developing T2D and other chronic diseases [9,20,24]. A longitudinal study reported that non-vegetarian middle-aged and older adults (aged 45–88 years) who consumed meat ≥1 time per week were 29% more likely to develop T2D compared to non-meat eaters over 17 years. Consumers of processed meats were 38% more likely to develop T2D [22]. Similarly, the Rotterdam study (*n* = 6798) found that the risk of developing T2D was lower in adult participants who followed PBD [25]. The participants with diets demonstrating the highest adherence to an overall PBD were associated with the lowest risk of developing T2D [25].

Whilst research investigating the benefits of PBD interventions has several positive indications, there are some limitations to the current research. Many of the studies have been conducted in specific populations [20,22,23], presenting difficulties in generalizing the findings. The Adventist Health Studies which reported significantly higher mortality and incidence rates of T2D in non-vegetarians were all carried out in the Seventh Day Adventist communities. These communities have distinct lifestyle behaviors that may also be contributing to these observations such as avoiding tobacco, caffeine, and alcohol as well as mostly adhering to vegetarian diets, thus translations of these findings may not reflect the general population [16,23]. Establishing a clear and concise definition of PBD and the categories within that classification is required as current definitions are inconsistent. Mihrshahi et al.’s Australian population-based study identified three categories of PBD including complete vegetarians, semi-vegetarians, and pesco-vegetarians but was unable to define further categories including vegans or lacto-ovo vegetarians because the study methodology did not initially target the identification of these dietary patterns [18].

Although the prevalence of T2D in Australian women is similar to that of men, the incidence rates for insulin dependent T2D are 1.7 times higher for females than males [4]. It has also been shown that women are more likely than males to consider a plant-based dietary pattern [14,18], and older adults that do follow PBD are more likely to choose to do so for health reasons [26]. Moreover, less is known about PBD in the Australian context and Australia is lagging with regard to dietary guidelines around plant-forward dietary patterns. Therefore, the current study aimed to describe the prevalence of IGT and DM among individuals following various PBDs and a meat-eating diet in a representative sample of older Australian women. The secondary aim is to examine the prevalence of IGT and DM across low and high frequencies of meat and processed meat consumption in a large representative sample of older Australian women.

## 2. Materials and Methods

### 2.1. The Australian Longitudinal Study on Women’s Health (ALSWH)

The ALSWH is a population-based study that examines the biological, physiological, social, and environmental determinants of health that impact women. Baseline data collection commenced in 1996 and subsequent surveys occurred at 3-year intervals. Three age cohorts were identified: young (1973–78), middle (1946–51), and old (1921–26). Participants were randomly selected using the Medicare database which contains names and addresses of all Australian citizens and permanent residents and 40 000 women initially agreed to participate in the project [27]. Random and remote women were sampled at twice the rate of urban areas so that these women were represented in a group large enough for statistical analysis. Comparison with census data indicates that the samples are reasonably representative of Australian women in the selected age groups [28]. This study was conducted according to the guidelines laid down in the Declaration of Helsinki and all procedures involving research study participants were approved by the University of Newcastle (approval number H-076-0795) and the University of Queensland (approval number 200400224). Written informed consent was obtained from all subjects. Further details of the ALSWH cohort have been reported elsewhere [28,29].

The current study is a cross-sectional analysis of data from survey 7 of the middle-aged cohort of the ALSWH. Survey 7 was conducted in 2013 when women were aged 62–67 years and this survey was selected because it is the most recent survey that collected dietary information. Women were included in the current analysis if they completed the food frequency questionnaire (FFQ) as part of survey 7. Participants with missing FFQ data as well as anyone with discrepancies between pictorial representations of meat serving sizes and frequency of meat consumption responses were excluded from the analysis as categorisation of their meat-eating status could not be conducted confidently, i.e., individuals who answered ‘never’ for all meat consumption questions but then when asked in a later question ‘When you ate steak did you usually eat?’ and selected a pictorial portion size for steak in the FFQ.

### 2.2. Plant-Based Dietary Patterns

Dietary intake was assessed using the Dietary Questionnaire for Epidemiological Studies (DQES) Version 2 which was administered as part of Survey 7. The DQES asked participants about the usual consumption of 74 foods and beverages and an additional 6 alcoholic beverages over the previous 12 months. Responses were recorded using a 10-point frequency scale ranging from “never” to “3 or more times per day”. Portion photographs of vegetables, meat, and casseroles were provided to assist participants with quantifying amounts consumed. Additional questions were asked about the frequency of consumption of items including cereals, sweets, snacks, meat, fruit, bread, eggs, spreads, and vegetables. The development and validation of the FFQ in a sample of Australian women has been reported elsewhere [30].

For the current study, dietary patterns were categorized according to the methods employed by Ferguson et al. [31] which implemented a modified and expanded version of definitions used by Mirshahi et al. in the 45 and Up population-based cohort study [18]. Responses from the DQES for respective food (meat, fish, dairy products, eggs, fat spreads) and beverage (dairy products) intake were used to define PBD and regular meat eaters. Responses from the DQES were converted to weekly equivalents by assigning scores to each frequency category, with ‘1 time per week’ receiving a score of one, and the remaining responses calculated as a factor of one (Table 1).

### 2.3. Frequency of Meat Consumption

Frequency of meat intake was defined across four categories: never, i.e., never eat meat; ≤1 time per week; >1 time per week but ≤2 times per week; >2 times per week but less than daily and lastly, daily or multiple times per day. Meat intake was further explored by examining the effect of processed meat consumption. The categories of processed meat in the FFQ include “*bacon*”, “*ham*”, “*corned beef, luncheon meats or salami*” and “*sausages or frankfurters”*. For consistency, weekly frequency of processed meat intake was also classified into weekly “low” and “high” consumption categories following the same categorization method described for low vs. high meat intake.

### 2.4. Impaired Glucose Tolerance and Diabetes

The outcomes of interest for this study were self-reported diagnosis and/or treatment for IGT and DM. In survey 7 participants were asked, “Have you been diagnosed or treated for: Diabetes (high blood sugar); impaired glucose tolerance, or none of these conditions”. Respondents were instructed to mark all options that applied to them in the past 3 years. Their responses defined four categories of data for analysis; diabetes; IGT; DM and IGT, and neither. Self-reported data for DM in the middle-aged cohort has previously been validated in the ALSWH using hospital administration records and the Medicare and Pharmaceutical Benefits Scheme databases [32].

### 2.5. Covariates

Data collected from survey 7 included information relating to demographic and socio-economic factors. Weight, height, and waist circumference were self-reported by participants. BMI was derived from weight (kg) divided by height (m^2^) and then categorized as underweight (<18.5 kg/m^2^); normal (18.5–24.9 kg/m^2^); overweight (25–30 kg/m^2^) and obese (˃30 kg/m^2^) as per the World Health Organization’s BMI classifications for adults [33]. Lifestyle factors identified as potential confounders when assessing the prevalence of DM and IGT across diet categories included physical activity levels, smoking status, use of certain dietary supplements (Coenzyme Q10 (CoQ10), Vitamin D, Fish oils), hormone replacement therapy (HRT), and alcohol intake [5,6,9,10,11]. Participants were asked validated questions from The Active Australia Survey to assess physical activity levels [34]. Responses indicated both the frequency and duration of exercise using a low to vigorous scale for intensity. Metabolic equivalents per week (minutes per week) were then assigned to each category of physical activity: Nil/sedentary; (0–<40); low (40–<600); moderate (600–<1200) and high (≥1200) [35]. Frequency of alcohol consumption was categorized in the ALSWH based on associated risk in accordance with the National Health and Medical Research Council (NHMRC) guidelines [36]. For the current study, this was condensed and summarized as “never”, “1–4 drinks/day”, and “≥5 drinks/day”. Smoking status was reported as “never smoked”, “ex-smoker”, “current smoker (<10 cigarettes/day)”, “current smoker (10–19 cigarettes/day)”, “current smoker (˃20 cigarettes/day)” and “indeterminate smoker (reported smoking but amount not provided)” [36,37]. In the current study smoking status has also been condensed and frequency summarised as ‘not at all’, ‘less than weekly’, ‘weekly’, and ‘daily’.

### 2.6. Statistical Analysis

Distributions of continuous variables were inspected using histogram plots and where normal are reported as mean ± standard deviation (SD). Comparisons were conducted using one-way Analysis of variance (ANOVA) with Tukey’s post hoc analysis to compare differences in continuous data such as anthropometry and age data between dietary categories. Continuous variables with skewed distributions are reported using median and interquartile range (IQR) and compared using the Kruskal–Wallis test. Categorical variables are presented as counts (n) and percentages (%) and categorical proportions were examined using the Pearson Chi-squared test. To examine the crude association between PBD, frequency of meat consumption, and the prevalence of IGT and DM, a binary logistic regression was performed. Multiple logistic regression was employed to adjust for potential confounding factors including BMI, physical activity levels, smoking status, dietary supplementation with Vitamin D, fish oils and CoQ10, HRT, and habitual alcohol intake. Odds ratios and 95% confidence intervals were reported where relevant. All analyses were conducted with the software package StataCorp 2016 (Stata Statistical Software: Release 14.2 College Station, TX, USA: StataCorp LP).

## 3. Results

### 3.1. Characteristics of Study Population

**Figure 1:**
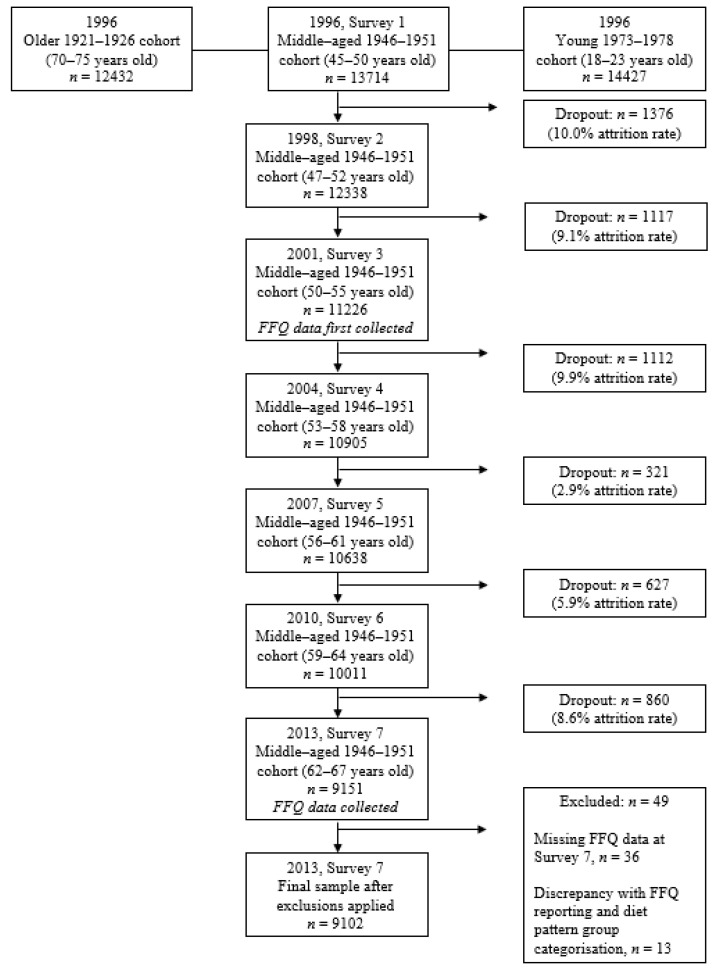
Australian Longitudinal Study of Women’s Health: flowchart of the 1946–51 cohort subjects.

A total of 9102 women who completed survey 7 (in 2013) of the ALSWH were included in the analysis. Of these, *n* = 49 participants were excluded who had incomplete FFQ data (*n* = 36) or had discrepancies in their responses about meat consumption and portion size (*n* = 13). Participant flow is seen in Figure 1 and has been reported previously [31].

These participants were excluded due to an inability to confidently categorize their meat-eating status. Table 2 presents the demographic, health, and lifestyle characteristics of participants across the 5 identified dietary patterns (vegan, lacto-ovo vegetarians, pesco-vegetarians, semi-vegetarians, and regular meat eaters). The mean age of the included participants was 64.3 years with most participants residing in major cities 38.5% (*n* = 3490) or inner regional areas 39.8% (*n* = 3609). Of the total respondents, 98.1% (*n* = 8927) were classified as regular meat eaters, 0.1% (*n* = 8) as vegans, 0.5% (*n* = 48) as lacto-ovo vegetarians, 0.8% (*n* = 74) pesco-vegetarians and 0.5% (*n* = 45) as semi-vegetarians. Supplement intake varied across the dietary patterns with pesco-vegetarians having a significantly higher intake of fish oils 57.5% (*n* = 42) and vitamin D 45.2% (*n* = 33) compared to regular meat eaters (47.2% and 34.7% respectively). CoQ10 usage was higher in all PBD groups (11.4% to 14.3% (*n* = 21) when compared with regular meat eaters 5.1% (*n* = 443). Compared with regular meat eaters, women who were categorized as vegan, lacto-ovo, pesco- or semi-vegetarians were less likely to be overweight or obese, less likely to consume alcohol, and less likely to be physically inactive. Vegan, lacto-ovo, and pesco-vegetarians were also less likely to smoke compared to regular meat eaters.

### 3.2. Prevalence of IGT and Diabetes across Dietary Patterns

In the total sample, 3.0% (*n* = 277) indicated they have been diagnosed or treated for IGT and 9.0% (*n* = 811) for DM in the last 3 years (Table 2). The requirement of treatment and/or diagnosis for DM was indicated by 9.1% (*n* = 800) of the regular meat eaters whilst IGT was indicated by 3.1% (*n* = 275) of the regular meat eaters. Vegan, lacto-ovo-, pesco- and semi-vegetarians reported lower rates of diagnosis and treatment for IGT compared to regular meat eaters. There were no respondents in the vegan or pesco-vegetarian dietary pattern groups that indicated they required treatment for or were diagnosed with diabetes in the 3 years prior. Lacto-ovo vegetarians and semi-vegetarians demonstrated a higher prevalence of diabetes compared to other PBD patterns (Table 2).

Since the prevalence of IGT and DM was scarce among the PBD groups in this cohort, for further exploration the PBD groups were consolidated into a single group “Vegetarians” which consisted of all vegans, lacto-ovo- and pesco-vegetarians. Semi-vegetarians and regular meat eaters were categorized as “All Meat Eaters”. A higher prevalence of DM was observed in All Meat Eaters (*n* = 806, 9.1%) compared with Vegetarians (*n* = 5, 3.9%) (Table 3).

### 3.3. Prevalence of IGT and Diabetes across Frequency of Meat Intake in All Meat Eaters

**Figure 2:**
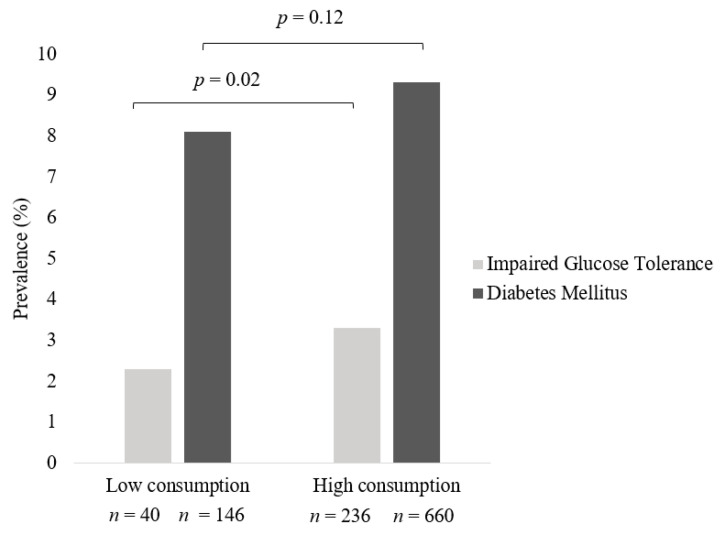
Prevalence of IGT and Diabetes across frequency of meat intake in All Meat Eaters in Australian women from the Australian Longitudinal Study on Women’s Health. Pearson chi-square test was used to compare prevalence of disease across groups. Data are presented as percentages. Low consumption encompasses individuals who consumed meat ≤1/week up to but less than daily and high consumption those who consumed meat daily/multiple times per day.

For ease of interpretation, meat intake categories were consolidated to compare “low” weekly consumption (consumed meat ≤1/week up to but less than daily) and “high” weekly consumption (consumed meat daily/multiple times per day). Women who consumed meat daily or multiple times per day had higher rates of IGT (*n* = 236, 3.3%) compared to those who consumed meat less frequently (*n* = 40, *p* = 0.02, 2.3%). Similarly, the prevalence of DM was higher in women who had a high weekly intake of meat (*n* = 660, 9.3%) compared to those who had a low intake of meat (*n* = 146, 8.1%); however, this was statistically non-significant (Figure 2).

### 3.4. Prevalence of IGT and Diabetes across Frequency of Processed Meat Intake in All Meat Eaters

**Figure 3:**
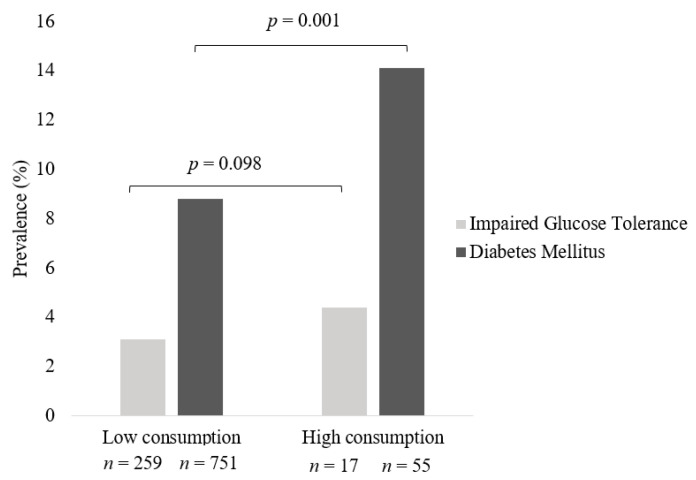
Prevalence of IGT and Diabetes across frequency of processed meat intake in All Meat Eaters in Australian women from the Australian Longitudinal Study on Women’s Health. Pearson chi-square test was used to compare prevalence of disease across groups. Data are presented as percentages. Low consumption encompasses individuals who consumed processed meat ≤1/week up to but less than daily and high consumption those who consumed processed meat daily/multiple times per day.

High weekly consumers of processed meats had a statistically significantly higher prevalence of DM of 14.1% (*n* = 55, *p* = 0.001) compared to low weekly consumers (*n* = 751, 8.8%) (Figure 3). Higher prevalence of treatment and or diagnosis for IGT (4.4%) was reported in individuals who were high consumers of processed meats compared to low consumers (3.1%), however, this observation did not reach statistical significance.

### 3.5. Odds of IGT and Diabetes in All Meat Eaters

Odds ratios for both the crude and adjusted models are presented in (Table 4). There was no significant association between high weekly consumption of meat and the odds of DM. Crude models revealed that individuals who had a high weekly consumption of meat had higher odds of IGT than those who consumed meat less frequently. The odds of DM were significantly higher in the group who had a high weekly frequency of processed meat intake compared to low intake, however, statistical significance was lost after adjusting for confounders, with this being largely due to BMI (*data not shown*). There was no significant relationship between high weekly frequency of processed meat consumption and IGT in either the crude or adjusted models (Table 4).

## 4. Discussion

The current study aimed to investigate the association of PBD with prevalence of IGT and DM in older Australian women. Descriptive cross-sectional analysis indicates that in a cohort of Australian women aged 62–67 years, women who consume a PBD are less likely to report a diagnosis of DM compared to women who regularly consume meat. This study also provided further insight into the relationship between frequency of all meat intake and processed meat intake and prevalence of IGT and DM. High weekly consumption of meat was associated with an increased risk of reporting IGT diagnosis, whilst a high weekly consumption of processed meat was associated with a significantly higher risk of reporting DM diagnosis in this cohort. After adjustment for covariates the statistical significance of these observations was lost, suggestive of the interplay between BMI, lifestyle characteristics and meat consumption and the prevalence of IGT and DM in this sample.

The prevalence of PBD groups and regular meat eaters in this cohort was similar to another large Australian population-based cohort study, “The 45 and Up Study” [18]. This study differed in its inclusion of both women and men across a broader age group (˃45 yrs), however, used similar PBD and meat eater categorizations. The prevalence of regular meat eaters was identical across both samples, but the prevalence of pesco-vegetarianism was lower (0.46%) and semi-vegetarianism was higher (0.82%) in “The 45 and Up” study compared to the current study. Internationally, studies investigating PBD have mostly reported higher prevalence rates than Australian studies [22,23,38]. The European Prospective Investigation into Cancer and Nutrition (EPIC)-Oxford study conducted with participants in the United Kingdom reported the prevalence of meat eaters (both low and high) at 50%, vegetarians, and vegans (including lacto-ovo vegetarians) at 34% and fish eaters at 16% [38]. Like the current study, the EPIC-Oxford study reported low prevalence rates of veganism, leading to the combined vegetarian and vegan categories. Discrepancies between study prevalence rates can somewhat be attributed to different reporting and categorization methods for PBDs. The diversity of cuisine globally may also be a significant contributing factor. The considerably higher rates of meat eaters in Australian based studies may be reflective of The Organization for Economic Co-Operation Development and Food and Agricultural Organization (OECD-FAO) report that showed Australia is consistently one of the highest consumers of meat in the world with an average of about 90 kg consumed annually per person [39].

Previous studies have reported varied findings on the efficacy of PBD to reduce the risk of developing IGT and DM [22,40,41]. The Adventist studies, some of the largest to investigate these links found lifelong vegetarian diets resulted in significantly lower rates of DM when compared to non-vegetarian diets. DM prevalence rates have been shown to increase incrementally across the vegetarian continuum: vegan (2.9%), lacto-ovo (3.2%), pesco-vegetarian (4.8%), semi-vegetarian (6.1%) and non-vegetarian (7.6%) groups [23,40] and this is similar to the trends in the current study. As previously mentioned, the Adventist studies do draw criticism, however, for not being representative of the general population [23].

Previous studies have reported that the higher the consumption of meat, the higher the risk of DM [42,43,44]. Findings from the current study concur with these reports, with low meat consumption associated with a lower reporting rate of IGT or DM diagnosis, but women identified as consuming meat daily or multiple times per day demonstrated a trend towards higher rates of IGT and DM. Epidemiological studies carried out in diverse populations indicate that the consumption of meat including red meat is related to an increased risk of developing T2D [42,43,44,45,46]. Conversely, pooled results from a recent systematic review and meta-analysis of 21 RCTs reported no significant impact of red meat intake on various glycaemic indices (i.e., insulin sensitivity, insulin resistance, fasting glucose, fasting insulin, glycated haemoglobin, pancreatic beta-cell function or glucagon-like peptide-1) [47].

The exact mechanistic pathways for the relationship between meat consumption and DM are still unknown but several components of red meat have been proposed. Findings from cross-sectional studies indicate an association between high saturated fatty acid (SFA) intake and decreased insulin sensitivity [44,45,46]. Decreased insulin sensitivity can be directly linked to IGT which the World Health Organization (WHO) identifies as a major risk factor for developing T2D [48]. It has also been argued that increased consumption of SFA may contribute to higher body weight and increased BMI, also significant risk factors for DM [31,44,47,48]. In the current study, regular meat eaters had a higher body weight, BMI, and waist circumference than all PBD groups. A systematic review and meta-analysis of RCTs reported that compared to regular meat diets, PBD interventions led to significantly lower body weight (−2.35 kg), BMI –0.90 kg/m^2^) and waist circumference (−4.23 cm) in individuals with T2D [19]. These findings support the potential implementation of PBDs for the management of central adiposity in individuals with T2D. Branched-chain amino acids (BCAA) another component of red meat, have also been positively associated with insulin resistance and T2D, with studies indicating elevated plasma concentration of BCAAs may impair insulin signaling activity [46,49,50]. Mammalian target of rapamycin (mTOR), heme iron, and advanced glycation end products (AGEs) are other dietary components that have been identified as plausible explanations for the elevated risk of DM in meat eaters [43,44,46,50]. Cohort studies also indicate that meat eaters engage in higher rates of unfavorable lifestyle behaviors compared to vegetarians [23,38,40]. Elevated BMI, smoking, alcohol consumption, and lower physical activity levels are all factors that had higher prevalence rates in the regular meat eater sample of this study and are also known to influence the risk of pre-diabetes/diabetes. After adjusting for characteristics, the odds of DM and high processed meat consumption and odds of IGT and high total meat consumption was lost and largely attributed to the addition of BMI to the model. Therefore, these findings provide evidence for the interplay between high meat consumption and unfavorable lifestyle behaviors amongst regular meat eaters, which is in line with previous studies conducted in other populations. Moreover, the influence of high total meat and processed meat consumption on risk of IGT and/or diabetes is likely mediated at least in part by BMI, which is consistent with our previous findings in this sample [31] and other studies [19]. Notably, normal margins of BMI for older Australian adults aged 65 years and over have been suggested, such that a healthy weight target of 24–30 kg/m^2^ may be more appropriate as mortality risk has been shown to be lowest at this BMI range [51,52]. Nevertheless, in the current study, regardless of BMI category we demonstrate that the relationship between meat and processed meat consumption and odds of reporting DM and IGT is attributed in part, to BMI. Future RCTs may provide further clarity and evidence for the association between meat consumption and the risk of IGT and DM as well as their interplay with other lifestyle factors and behaviors.

Our findings on the association between IGT/diabetes and total meat and processed meat consumption are supported by previous observational studies [42,46,53,54]. A recent meta-analysis of cohort studies including over 680,000 participants reported findings in similar agreement with the current study, whereby compared to the lowest intake group, high consumption of processed red meat and unprocessed red meat increased T2D risk by 27% and 15%, respectively [54]. A prospective study conducted in French women found that those consuming a daily amount of processed meat (48g) had a significantly higher incidence of DM than women consuming 5 g of processed meat <1/week [51]. The exact mechanisms underpinning this association are unclear, however, there are additional components in processed meats such as additives to extend shelf-life, enhance flavor, odor, texture, and appearance that in combination with the naturally occurring compounds may contribute to this unfavorable association with chronic disease. Processed meats can contain 40% more sodium and up to 50% more nitrates than unprocessed meats, both of which may have a negative impact on glucose regulation and metabolism [46,51]. AGEs which are present in red meats are found in considerably higher levels in processed meats (e.g., raw beef 707 kU/100 g vs. broiled beef frankfurter 11,270 kU/100 g) and have been reported to contribute to the pathogenesis of T2D [45,46]. Future studies are needed to better understand the mechanisms underlying processed meat consumption and the potential effect on T2D development.

A key strength of the current study is the large nationally representative sample of women drawn from the ALSWH. The ALSWH participant sample has been validated as broadly representative of the general Australian female population when compared with census data [28]. Using the middle-aged cohort of the ALSWH (born 1946–51) provided an opportunity to review a population with significant occurrence of the target conditions. Furthermore, definitions of PBD employed in this study are consistent with those used in other Australian population-based studies [18,31]. Some limitations have been identified within the current study. Whilst the PBD and regular meat eater categories in the current study are similar to other Australian based studies, it must be noted that the participant numbers in some of the PBD categories were scarce and therefore limited the ability to perform meaningful comparisons across each specific PBD group. The authors attempted to mediate this limitation by combining PBD groups under the one umbrella of ‘Vegetarian’ to allow for more meaningful statistical comparisons, in line with other studies who had this same limitation [18,55]. The lack of biomarker data in the ALSWH is a limitation of the current study, as biomarkers relevant to IGT and DM would provide objective insight into the relationship between dietary patterns explored and glycaemic conditions. All outcomes, exposures, and covariates in this study were self-reported. Whilst self-reported data can be problematic, it is important to note that numerous criteria in the ALSHW have been previously validated. Self-reported diabetic status in the ALSWH has been compared with The Australian Diabetes and Lifestyle Study and hospital records and was found to be an accurate reflection of DM prevalence in middle-aged Australian women [32]. The FFQ used throughout the ALSWH has also been previously validated [30]. Although the intake of meat and PBD was assessed through the validated FFQ the associations shown may not only be related to meat intake as other food groups were not explored in the current study. Future studies are warranted to further explore the association between IGT, DM and varying types of meat consumption as well as consider adjusting for other key nutrients such as energy, as this was outside the scope of the current study and thus a limiting factor. As with all observational studies, caution must be exercised when inferring causation from the results. Appropriate attempts were made to adjust and report for confounding lifestyle factors, however, further exploration of other dietary food groups and their association with PBD and IGT and DM are warranted in future studies.

## 5. Conclusions

The present study findings suggest an association between high consumption of meat and reported diagnosis of IGT and high consumption of processed meat and reported diagnosis of DM in a representative sample of older Australian women. In this study, these associations appear to be influenced at least in part by BMI, a known risk factor for DM. Further investigations into the biological mechanisms responsible for the observed associations are required. These findings highlight the need for future studies that explore the wider health implications of PBD and meat consumption in the broader Australian population to inform dietary guidelines around achieving nutritional adequacy and healthful PBD for reducing chronic disease risk.

## Figures and Tables

**Table 1:** Classification of diet groups by the number of time(s) foods were consumed per week ^1^.

	Vegan (*n* = 8)	Lacto-Ovo Vegetarian (*n* = 48)	Semi-Vegetarian (*n* = 45)	Pesco-Vegetarian (*n* = 74)	Regular Meat Eater (*n* = 8927)
Times per week consumed:					
Beef, veal, chicken, lamb, pork, bacon, ham, corned beef, luncheon meats or salami, sausages or frankfurters	0	0	≤1	0	0 or ≥1
Fish, steamed, grilled, or baked; fish, fried (include take-away), fish, tinned (salmon, tuna, sardines etc.)	0	0	0 or ≤1	≥1	0 or ≥1
Total of above categories	0	0	≤ 1	≥1	˃1
Usual eating habits ^2^					
Milk, cheese, ice-cream, yoghurt	Nil	Y	N/A	N/A	N/A
Butter, butter, and margarine blends	Nil	Y	N/A	N/A	N/A
Eggs	Nil	Y	N/A	N/A	N/A

Dietary patterns categorized according to defining characteristics employed by Ferguson et al. [31]. ^1^ DQES items were converted to weekly equivalents by assigning scores to each frequency category. With ‘1 time per week’ receiving a score of 1, and the remaining responses calculated as a factor of 1. ^2^ Only habitual intake (and not frequency) of these foods was required for classification of vegan and lacto-ovo vegetarian. Frequency on intake was not provided for butter and butter and margarine blends in the DQES.

**Table 2:** Characteristics of all study participants of the 1946–51 aged cohort of the Australian Longitudinal Study of Women’s Health at Survey 7 (in 2013). Data are presented as mean and standard deviation or counts and (percentages) unless otherwise specified.

	Vegan (*n* = 8)			Lacto-Ovo Vegetarian (*n* = 48)			Pesco-Vegetarian (*n* = 74)			Semi-Vegetarian (*n* = 45)			Regular Meat Eater (*n* = 8927)			Total (*n* = 9102)			
	Mean	SD	*n ^2^*	Mean	SD	*n*	Mean	SD	*n*	Mean	SD	*n*	Mean	SD	*n*	Mean	SD	*n*	*p* ^1^
Age (years)	64.4	1.8		64.1	1.5		64.3	1.5		64.6	1.5		64.3	1.5		64.3	1.5		0.78
Height (cm)	162.4	6.7		162.7	5.4		162.8	7.1		162.9	6.7		162.9	6.6	8916	162.9	6.6	9091	1.00
Weight (kg)	63.7	9.7		66.4	15.3	47	63.7	13.4	72	71.0	13.4	43	73.8	15.6	8629	73.7	15.6	8799	<0.001
WC (cm)	79.9	12.6	7	87.3	13.7	45	83.0	11.8	68	89.0	13.2	38	91.4	13.7	7980	91.3	13.7	8138	<0.001
BMI (kg/m^2)^	24.1	3.1		25.0	5.0	47	24.0	4.5	72	26.8	5.3	43	27.8	5.7	8620	27.8	5.7	8790	<0.001
Overweight or obese ^3^	2 (25.0)			21 (44.7)			25 (34.7)			27 (62.8)			5536 (64.2)			5611 (63.8)			<0.001
IGT ^4^	0 (0.0)			1 (2.17)			0 (0.0)			1 (2.2)			275 (3.1)			277 (3.0)			
Diabetes	0 (0.0)			5 (10.9)			0 (0.0)			6 (13.3)			800 (9.1)			811 (9.0)			
Residence																			<0.008
Major cities	5 (62.5)			23 (47.9)			35 (47.3)			19 (43.2)			3408 (38.3)			3490 (38.5)			
Inner regional	3 (37.5)			23 (47.9)			30 (40.5)			12 (27.3)			3541 (39.8)			3609 (39.8)			
Outer regional	0 (0.0)			1 (2.1)			8 (10.8)			11 (25.0)			1697 (19.1)			1717 (18.9)			
Remote	0 (0.0)			1 (2.1)			1 (1.4)			2(4.6)			254 (2.9)			258 (2.8)			
Employment																			0.096
Retired/never worked	4 (57.1)			21 (43.8)			37 (50.0)			29 (65.9)			5158 (58.8)			5249 (58.7)			
Not retired	3 (42.9)			27 (56.3)			37 (50.0)			15 (34.1)			3608 (41.2)			3690 (41.3)			
Smoking Status																			0.22
Not at all	8 (100.0)			46 (97.9)			69 (94.5)			40 (88.9)			8271 (93.2)			8434 (93.2)			
< weekly	0 (0.0)			1 (2.1)			1 (1.4)			1 (2.2)			43 (0.5)			46 (0.5)			
Weekly	0 (0.0)			0 (0.0)			0 (0.0)			0 (0.0)			37 (0.4)			37 (0.4)			
Daily	0 (0.0)			0 (0.0)			3 (4.1)			4 (8.9)			522 (5.9)			529 (5.8)			
Alcohol Intake																			<0.001
Never	5 (62.5)			14 (29.8)			22 (29.7)			22 (53.7)			1411 (16.3)			1474 (16.7)			
1–4/day	3 (37.5)			33 (70.2)			52 (70.3)			19 (46.3)			7156 (82.7)			7263 (82.3)			
˃5/day	0 (0.0)			0 (0.0)			0 (0.0)			0 (0.0)			86 (1.0)			86 (1.0)			
Supplement Intake ^5^																			
Fish Oils	1 (14.3)			11 (23.9)			42 (57.5)			13 (29.5)			4162 (47.2)			4229 (47.1)			<0.001
Vitamin D	1 (12.5)			18 (39.1)			33 (45.2)			13 (30.2)			3074 (34.7)			3139 (35.0)			0.22
CoQ10	1 (14.3)			6 (13.3)			9 (12.3)			5 (11.4)			443 (5.1)			464 (5.2)			0.001
HRT	0 (0.0)			2 (4.2)			10 (13.5)			2 (4.4)			832 (9.3)			846 (9.3)			0.36
Physical Activity (mins per week ^6^																			
	M	IQR	*n*	M	IQR	*n*	M	IQR	*n*	M	IQR	*n*	M	IQR	*n*	M	IQR	*n*	
Sedentary	100	(30,240)		165	(45,390)		180	(90,300)	73	75	(0,240)	42	120	(30,300)	8675	120	(30,300)	8846	0.062
Low	15	(0,150)		30	(0,130)		0	(0,90)	73	0	(0,30)	42	0	(0,120)	8735	0	(0,120)	8906	0.19
Moderate	0	(0,15)		0	(0,45)		0	(0,60)	73	0	(0,0)	41	0	(0,0)	8763	0	(0,0)	8933	0.072
High	150	(0,240)		125	(30,300)		120	(0,300)	73	60	(0,125)	41	150	(0,300)	8659	150	(0,300)	8829	0.042

SD, standard deviation; WC, waist circumference; IGT, impaired glucose tolerance; CoQ10, coenzyme Q10; HRT, Hormone replacement therapy; IQR, interquartile range; M, median. ^1^ *p*–values represent the level of significance for difference across groups and were obtained for normally distributed continuous data using ANOVA. Skewed continuous data were compared using Kruskal–Wallis and categorical data were compared using Fisher’s Exact test. ^2^ For measures with missing data that are not already presented as counts and percentages, the number of participants has been provided. ^3^ Overweight and obese categories are defined by the WHO recommendations for adults; overweight BMI ˃25 kg/m^2^ and <30 kg/m^2^ and obese ≥30 kg/m^2^. ^4^ IGT and diabetes status were self-reported as being diagnosed and/or requiring treatment in the past 3 years. ^5^ Dietary supplement use in the past 4 weeks. ^6^ Physical activity is presented as median (M) and interquartile range (IQR) for the minutes of activity undertaken in the last week *n* represents the number of individuals with available data when groups had data missing.

**Table 3:** Prevalence rates of IGT and diabetes in Vegetarian and All Meat Eaters groups. Data are presented as count and (percentage).

	Vegetarians ^1^ (*n* = 128)	All Meat Eaters ^2^ (*n* = 8875)	Total (*n* = 9003)
IGT	1(0.8)	276 (3.1)	277
Diabetes	5 (3.9)	806 (9.1)	811

^1^ Includes vegans, lacto-ovo- and pesco-vegetarians. ^2^ Includes semi-vegetarians and regular meat eaters.

**Table 4:** Crude and adjusted models of the odds of IGT and DM across meat and processed meat intake for the Total Sample and All Meat Eaters in the ALSWH 1946–51 cohort.

	Total Sample ^1^				All Meat Eaters ^2^			
	OR	SE	95% CI	*p*	OR	SE	95% CI	*p*
Frequency of all meat intake								
Diabetes Mellitus								
⋅Low intake of meat	1.0	-	reference	-	1.0	-	reference	-
⋅High intake of meat—Crude model	1.2	0.1	1.0, 1.4	0.06	1.2	0.1	1.0, 1.4	0.12
⋅High intake of meat—Adjusted model	1.1	0.1	0.9, 1.4	0.28	1.1	0.1	0.9, 1.4	0.37
Impaired Glucose Tolerance								
⋅Low intake of meat	1.0	-	reference	-	1.0	-	reference	-
⋅High intake of meat—Crude model	1.6	0.3	1.1, 2.2	0.01	1.5	0.3	1.1, 2.1	0.02
⋅High intake of meat—Adjusted model	1.4	0.3	1.0, 2.0	0.07	1.4	0.3	0.9, 2.0	0.10
Frequency of processed meat intake								
Diabetes Mellitus								
⋅Low intake of processed meat	1.0	-	reference	-	1.0	-	reference	-
⋅High intake of processed meat—Crude model	1.7	0.3	1.3, 2.3	<0.0001	1.7	0.3	1.3, 2.3	<0.0001
⋅High intake of processed meat—Adjusted model	1.2	0.2	0.8, 1.7	0.44	1.1	0.2	0.8, 1.7	0.46
Impaired Glucose Tolerance								
⋅Low intake of processed meat	1.0	-	reference	-	1.0	-	reference	-
⋅High intake of processed meat—Crude model	1.5	0.4	0.9, 2.4	0.13	1.5	0.4	0.9, 2.4	0.15
⋅High intake of processed meat—Adjusted model	1.1	0.3	0.6, 2.0	0.80	1.1	0.3	0.6, 2.0	0.281

Data are presented as odds ratios, standard errors, *p*-values, and 95% confidence interval. ^1^ Total sample (*n* = 9102). ^2^ All Meat Eaters includes participants who identified as regular meat eaters and semi-vegetarians (*n* = 8875). CI, confidence interval; OR, odds ratio; SE, standard error.

## Data Availability

The data that support the findings of this study are available from Australian Longitudinal Study on Women’s Health, but restrictions apply to the availability of these data, which were used under license for the current study, and so are not publicly available. Data are however available from the authors upon reasonable request and with permission of Australian Longitudinal Study on Women’s Health.
